# A yeast three-hybrid system that reconstitutes mammalian hypoxia inducible factor regulatory machinery

**DOI:** 10.1186/1471-2121-9-18

**Published:** 2008-04-10

**Authors:** Maria L Alcaide-German, Alicia Vara-Vega, Luis F Garcia-Fernandez, Manuel O Landazuri, Luis del Peso

**Affiliations:** 1 Departamento de Bioquímica, Instituto de Investigaciones Biomédicas 'Alberto Sols', Consejo Superior de Investigaciones Científicas, Universidad Autónoma de Madrid, Arturo Duperier 4, 28029 Madrid, Spain; 2 Servicio de Inmunología, Hospital de la Princesa-UAM, Diego de León, 62. 28006 Madrid, Spain; 3 R&D Department, PharmaMar, S.A.PharmaMar, Avenida de los reyes, 1. E-28770 Colmenar Viejo, Spain

## Abstract

**Background:**

Several human pathologies, including neoplasia and ischemic cardiovascular diseases, course with an unbalance between oxygen supply and demand (hypoxia). Cells within hypoxic regions respond with the induction of a specific genetic program, under the control of the Hypoxia Inducible Factor (HIF), that mediates their adaptation to the lack of oxygen. The activity of HIF is mainly regulated by the EGL-nine homolog (EGLN) enzymes that hydroxylate the alpha subunit of this transcription factor in an oxygen-dependent reaction. Hydroxylated HIF is then recognized and ubiquitinilated by the product of the tumor suppressor gene, pVHL, leading to its proteosomal degradation. Under hypoxia, the hydroxylation of HIF by the EGLNs is compromised due to the lack of oxygen, which is a reaction cosubstrate. Thus, HIF escapes degradation and drives the transcription of its target genes. Since the progression of the aforementioned pathologies might be influenced by activation of HIF-target genes, development of small molecules with the ability to interfere with the HIF-regulatory machinery is of great interest.

**Results:**

Herein we describe a yeast three-hybrid system that reconstitutes mammalian HIF regulation by the EGLNs and VHL. In this system, yeast growth, under specific nutrient restrictions, is driven by the interaction between the β domain of VHL and a hydroxyproline-containing HIFα peptide. In turn, this interaction is strictly dependent on EGLN activity that hydroxylates the HIFα peptide. Importantly, this system accurately preserves the specificity of the hydroxylation reaction toward specific substrates. We propose that this system, in combination with a matched control, can be used as a simple and inexpensive assay to identify molecules that specifically modulate EGLN activity. As a proof of principle we show that two known EGLN inhibitors, dimethyloxaloylglycine (DMOG) and 6-chlor-3-hydroxychinolin-2-carbonic acid-N-carboxymethylamide (S956711), have a profound and specific effect on the yeast HIF/EGLN/VHL system.

**Conclusion:**

The system described in this work accurately reconstitutes HIF regulation while preserving EGLN substrate specificity. Thus, it is a valuable tool to study HIF regulation, and particularly EGLN biochemistry, in a cellular context. In addition, we demonstrate that this system can be used to identify specific inhibitors of the EGLN enzymes.

## Background

Most mammalian tissues are strictly dependent on oxidative metabolism and require constant oxygen supply to maintain cell function. Thus, acute oxygen deprivation, even for brief periods of time, can be deleterious. In contrast, cells can adapt to moderate chronic hypoxia through the induction of a specific gene expression program. The induction of this set of genes adjusts the metabolism by increasing anaerobic glycolysis [1] and fine-tuning mitochondrial respiration [2,3]. In addition, hypoxic cells induce vascularization of the poorly oxygenated tissue through the expression of proangiogenic molecules such as VEGF [4]. The family of Hypoxia Inducible Factors (HIFs) is responsible for the induction of the vast majority of the genes involved in these adaptive responses [5,6]. HIF transcription factors are heterodimers of an oxygen dependent alpha subunit (HIFα) and a beta (HIFβ) chain that is shared with other transcription factors of the basic-helix-loop-helix family. There are three different HIFα isoforms (HIF1α, HIF 2α and HIF 3α), encoded by independent genes, whose regulation by oxygen is thought to be similar. Oxygen affects the half life [7,8] as well as the transcriptional activity [9] of HIFα proteins. In both cases, the effect is mediated by hydroxylation of specific residues, within HIFα, by a family of 2-oxogluatarate-dependent dioxygenases that require molecular oxygen as cosubstrate. These modifications affect the ability of HIF to interact with other proteins. Specifically, hydroxylation of a conserved asparagine residue (N803 in human HIF1α protein) within the C-terminal transactivation domain by the Factor Inhibiting HIF (FIH) [10,11] prevents its interaction with the p300 coactivator [9]. On the other hand, the activity of the EGL-nine homologues (EGLNs, [12]) results in the hydroxylation of two conserved proline residues (P402 and P564 in human HIF1α) within the Oxygen-Dependent Degradation domain (ODD) of HIFα proteins[13]. Proline hydroxylation allows the binding of the E3 ubiquitin ligase pVHL to HIFα leading to its ubiquitination and proteosomal degradation [8,14,15]. Since FIH and EGLNs require oxygen as cosubstrate, under hypoxia their activity is reduced [16,17]. As a consequence the interaction with pVHL is lost, leading to HIF stabilization, while the interaction with p300 is allowed resulting in enhanced transcriptional activity. Although the regulation of HIF transcriptional activity seems to be critical for the induction of some HIF-target genes during hypoxia [18], the major effect of oxygen is the regulation of HIFα stability through the activity of the EGLNs. To date three independent enzymes termed EGLN1, 2 and 3 (also known as PHD2, 1 and 3 respectively) have been described to mediate HIFα proline hydroxylation [7,19,20]. Intriguingly, these enzymes display substrate specificity: while EGLN1 and 2 are capable of hydroxylating both HIFα prolines, EGLN3 is unable to act upon the N-terminal residue (P402 in human HIF1α) [17,21-23]. In this regard, recent publications describe novel potential targets for these enzymes that seem to be regulated by specific isoforms [24,25].

In addition to the relevance of this pathway in the physiological response to reduced oxygen supply, it probably plays a major role during the progression of several pathologies that course with hypoxia. Specifically, activation of HIF has been implicated in the induction of the glycolitic and angiogenic phenotype of cancer [4,26], generation and progression of tumors [27] and resistance to the isquemic insults (preconditioning) [28,29] among other phenomena. Thus, the identification of small molecules that modulate this pathway is of great therapeutical interest. In fact, several molecules with the ability to modulate HIF activity have been already described. In animal models, HIF inhibitors have been proven useful in the treatment of tumors [30], while molecules with the ability to induce HIF result in angiogenesis *in vivo *[31].

In this work, we describe the generation of a three-hybrid system that accurately reconstitutes mammalian HIFα regulation by EGLN hydroxylation and subsequent VHL binding. This system can be of aid in the study of EGLN biochemistry and the characterization of novel substrates. In addition, we demonstrate that it can be used as an efficient assay to screen for small molecules with the ability to regulate HIF.

## Results

### Generation of a yeast three hybrid-based system that reconstitutes HIF regulation

**Figure 1:**
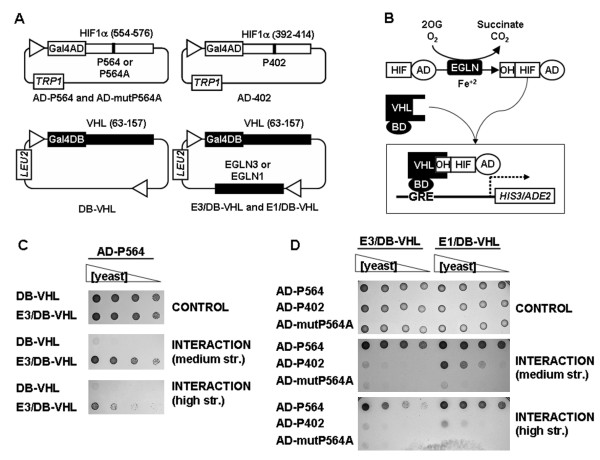
**Characterization of a yeast three-hybrid system that reconstitutes HIF regulatory machinery**. A, Schematic diagram representing the yeast three-hybrid constructs. Numbers in brackets correspond to residue positions for the encoded proteins. *TRP1 *and *LEU2*, genes encoding for the enzymes that allow cell growth in the absence of Trp and Leu respectively. B, Schematic diagram of the interaction between the different system components. 2OG, 2-oxoglutarate. C and D, Serial dilutions of clones transformed with the indicated constructs were grown on plates lacking Leu and Trp (CONTROL), plates lacking Leu, Trp and His (INTERACTION, medium str.) or plates lacking Leu, Trp, His and adenine (INTERACTION, high str.). The results shown are representative of at least three independent experiments.

Several EGLN assays have been described which include the determination of enzyme activity by measure of cosubstrates (dioxygen and 2-oxoglutarate) consumption/CO_2 _production and the detection of HIF hydroxylation using mass spectrometry or capture of VHL [32,33]. In addition, strategies to determine EGLN activity within cells have also been developed. These include the detection of reporter proteins fused to ODD [34] and the use of antibodies raised against hydroxyproline-containing ODDs [35]. The major limitation of the *in vitro *assays is that they are performed in a non physiological environment. On the other hand, the cell-based assays have to deal with the endogenous HIF machinery. To circumvent these problems we sought to reconstitute the HIF regulatory machinery in yeast cells, where expressed proteins are in a cellular environment but where no endogenous proteins related to the HIF pathway are expressed, therefore eliminating possible sources of interference. To this end, we cloned a fragment of human HIF1α (residues 554–576) containing P564 in frame with yeast Gal4 activation domain (AD-P564) (figure 1A). This fragment is sufficient for EGLN recognition [23] and VHL binding [36]. In addition, we cloned a VHL fragment encoding for its β-domain (residues 63 to 157) in frame with the yeast Gal4 DNA binding domain (DB-VHL) (figure 1A). The VHL β-domain is involved in HIF1α binding, while the α-domain, missing in this construct, interacts with the elongins B/C that are required to promote HIFα ubiquitination. Then we tested the interaction between these fusion proteins as their ability to support the growth of a yeast strain conditionally auxotrophic for histidine and adenine (figure 1B). As shown in figure 1C, yeast cells transformed with AD-P564 in combination with DB-VHL failed to grow in plates lacking histidine, suggesting lack of significant interaction between the two fusion proteins. This result was expected since no EGLN orthologs are present in yeast and VHL binding to HIF1α is strictly dependent on proline hydroxylation [8,14]. In addition, these data indicates that residual binding of VHL to non-hydroxylated AD-P564 is below detection limit in our assay conditions. Importantly, the expression of EGLN3 (construct E3/DB-VHL, figure 1A), together with the two fusion proteins, is sufficient to promote direct binding of DB-VHL to AD-P564, as indicated by the growth of transformed yeast in restrictive plates (figure 1C). The effect was not restricted to EGLN3 since the expression of EGLN1 also triggered the interaction between the fusion proteins (figure 1D). To further confirm that DB-VHL/AD-P564 interaction was mediated by the hydroxylation of AD-P564 by EGLNs, we repeated the experiment using a mutant HIF peptide in which P564 was replaced by an alanine residue (AD-mutP564A, figure 1A). As shown in figure 1D, DB-VHL was unable to interact with AD-mutP564A regardless of the presence of either EGLN. Moreover, in spite of relaxed target requirements [23,37], it has been described that EGLN isoforms display a specific preference for the two target sequences in HIF1α [17,21-23]. Therefore, we next wanted to determine whether in this system the EGLNs retained their native substrate specificity. Figure 1D shows that EGLN3 efficiently hydroxylates the peptide containing the sequence surrounding proline 564 (AD-P564), but it has no detectable activity toward a peptide comprising residues 392–414 derived from HIF1α (AD-P402). In contrast, EGLN1 was able to hydroxylate both sequences allowing their binding to VHL (figure 1D). Interestingly, the activity of EGLN1 toward AD-P402 was slightly lower than its activity using AD-P564 substrate as evidenced by the different growth of yeast transformed with each sequence in the high stringency plates (figure 1D). These results are in perfect agreement with previous reports describing the activity of the EGLN isoforms against different substrates [23,37-39]. Finally, further confirmation that EGLNs retained their substrate specificity was obtained through the analysis of the effect of L574A and L562R mutations (Additional file 1).

All together, these results indicate that this system faithfully reconstitutes the regulation of HIFα proteins by the sequential action of EGLN enzymes and VHL binding. In addition, data show that the β-domain of VHL is sufficient for target recognition and binding.

### The reconstituted HIF/EGLN/VHL system can be exploited to identify specific EGLN inhibitors

**Figure 2:**
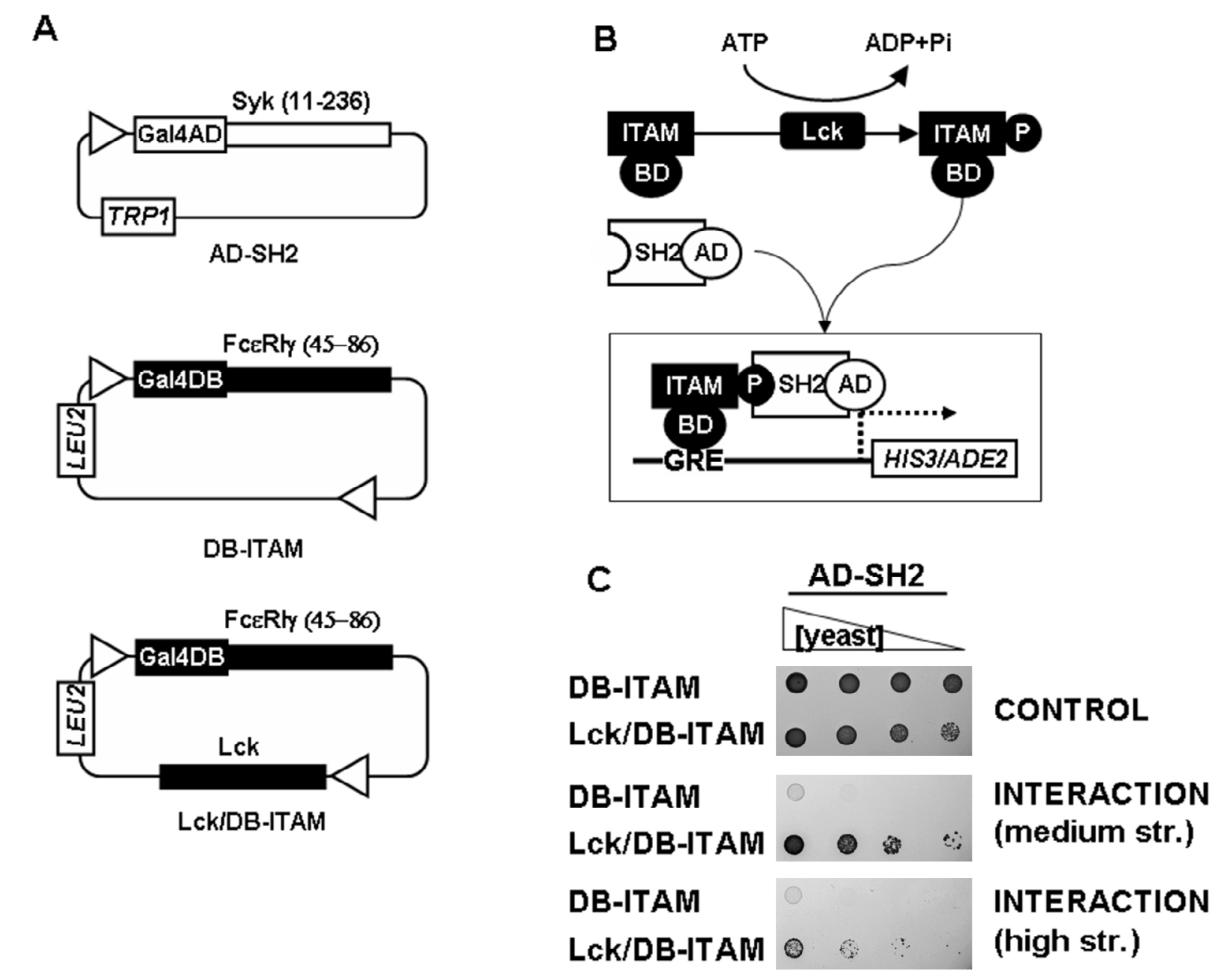
**Characterization of a control yeast three-hybrid system based on an ITAM/SH2 interaction that depends on Lck activity**. A, Schematic diagram representing the constructs. Abbreviations are as in figure 1. B, Schematic diagram of the interaction between the different system components. C, Serial dilutions of clones transformed with the indicated constructs were grown on different astringency plates. Symbols, abbreviations and panel labels are as in figure 1. The results shown are representative of at least three independent experiments.

The system described above relies on EGLN activity for DB-VHL/AD-P564 interaction, thus we reasoned that EGLN inhibitors should interfere with yeast growth. However, in cell-based assays, the inhibitory effects of drugs have to be carefully controlled for unspecific side effects on cell physiology or on the assay system itself. In order to have a control for the drug specificity and rule out potential side effects on yeast growth, we generated a set of constructs analogous to those described above but based on a protein machinery unrelated to the HIF pathway. In this control system, yeast growth is dependent on the interaction between an immunoreceptor tyrosine-based activation motif (ITAM), derived from the type Iγ IgE receptor (FcεRIγ), and the Src-Homology domain 2 (SH2) from the Syk protein. Importantly, the binding of Syk-SH2 tandem domains to the FcεRIγ-ITAM is dependent on the tyrosine phosphorylation of the latter [40]. The vectors generated for this system (figure 2A) encode for a Syk-derived tandem SH2 domains fused to the activation domain of Gal4 (AD-SH2) and for an ITAM from FcεRIγ fused to the Gal4 DNA binding domain (DB-ITAM). Finally, the cytoplasmic tyrosine kinase Lck was cloned into the pBridge vector to induce DB-ITAM phosphorylation and trigger its binding to AD-SH2 (figure 2B). Since yeast do not contain orthologues of the proteins of this system, AD-SH2 did not significantly interact with DB-ITAM unless the tyrosine kinase Lck was co-expressed in the assay (figure 2C), as previously reported [40]. Thus, the Lck and EGLN-based assays are analogous in that both have three components: an enzyme and two fusion proteins whose interaction allows yeast growth in restrictive media. Additionally, in both systems the interaction between the fusion proteins, and thus yeast growth, requires a modification of one of these proteins by the enzyme (compare figures 1B and 2B).

**Figure 3:**
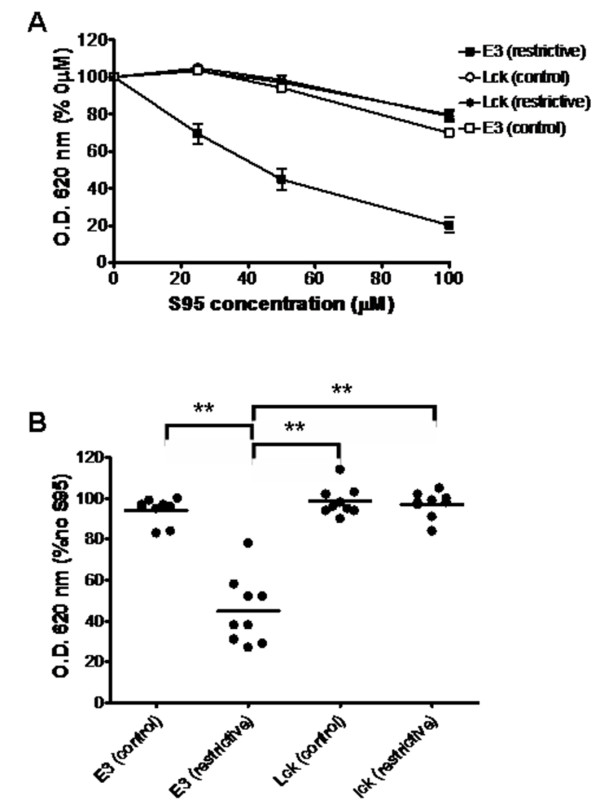
**Effect of S956711 on the EGLN-dependent and Lck-dependent three-hybrid systems**. Yeast clones transformed with constructs encoding for AD-P564 and E3/DB-VHL (E3) or AD-SH2 and Lck/DB-ITAM (Lck) were used to inoculate control media lacking Leu and Trp (control) or restrictive media (restrictive) lacking Leu, Trp, and His (in the case of E3 clones, restrictive media also lacked adenine). Duplicate cell cultures were grown in the presence of 0, 25, 50 or 100 μM S956711. The density of cultures was determined 16–24 hours after inoculation. A, The cell density (absorbance at 620 nm) of each culture condition is represented as the percentage of density obtained for the cells grown in the absence of drug. The graph represents the average values of nine independent experiments. Error bars represent the S.E.M. B, the individual data values of density for the 50 μM S956711 cultures in A are represented. Horizontal bars represent the mean. Statistically significant different mean values (p < 0.001) are indicated with asterisks.

**Figure 4:**
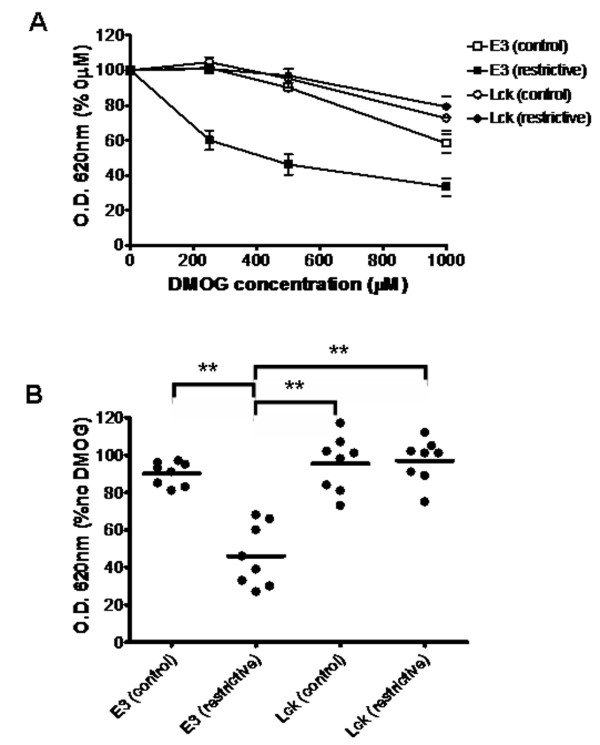
**Effect of DMOG on the EGLN-dependent and Lck-dependent three-hybrid systems**. Yeast clones transformed with constructs encoding for AD-P564 and E3/DB-VHL (E3) or AD-SH2 and Lck/DB-ITAM (Lck) were used to inoculate control media lacking Leu and Trp (control) or restrictive media (restrictive) lacking Leu, Trp, and His (in the case of E3, clones restrictive media also lacked adenine). Duplicate cell cultures were grown in the presence of 0, 250, 500 or 1000 μM DMOG. The density of cultures was determined 16–24 hours after inoculation. A, The cell density (absorbance at 620 nm) of each culture condition is represented as the percentage of density obtained for the cells grown in the absence of drug. The graph represents the average values of eight independent experiments. Error bars represent the S.E.M. B, the individual data values of density for the 500 μM DMOG cultures in A are represented. Horizontal bars represent the mean. Statistically significant different mean values (p < 0.001) are indicated with asterisks.

Next we tested the ability of these combined systems to detect molecules that interfere with HIF regulation. For this purpose we selected two previously characterized EGLN inhibitors, dimethyloxaloylglycine (DMOG) [41] and 6-chlor-3-hydroxychinolin-2-carbonic acid-N-carboxymethyl-amide (S956711) [31]. In order to facilitate the quantification of the effects of inhibitors on these systems, we set up the experiments in liquid media so that yeast growth could readily be determined by the optical density of the cell culture. Since the growth rate of yeast transformed with different constructs in each culture media varies, we adjusted the initial concentration of each yeast strain for each media so that all cultures achieved logarithmic growth in overlapping time windows (Additional file 2). As shown in figure 3A, treatment with increasing doses of S956711 had a strong effect on the growth of yeast expressing AD-HIF and DB-VHL/EGLN3 in restrictive media. In contrast, when these cells were cultured in control media, where no interaction between fusion proteins was required for growth, S956711 had only a minor effect when used at 100 μM (figure 3A). Thus, the effect of S956711 is not due to an unspecific effect on yeast viability/growth. To further confirm the specificity of the effect upon the HIF system, we tested the effect of S956711 on yeast transformed with AD-SH2 and DB-ITAM/Lck. As shown in figure 3, S956711 had no significant effect in this control system regardless of the culture media. These results rule out that the inhibition of the EGLN system could be due to side effects of S956711 on transcription/translation of the three-hybrid elements. The specific inhibitory effect of S956711 was highly reproducible and unspecific effects were undetectable at doses below 100 μM (figure 3B). Similarly to the results observed for S956711, treatment with DMOG also resulted in a dose-dependent inhibition of the interaction between AD-HIF and DB-VHL (figure 4A). As shown for S956711, DMOG inhibition was specific for the HIF regulatory machinery, since no effect was observed on the binding of AD-SH2 to DB-ITAM (figures 4A and 4B).

**Figure 5:**
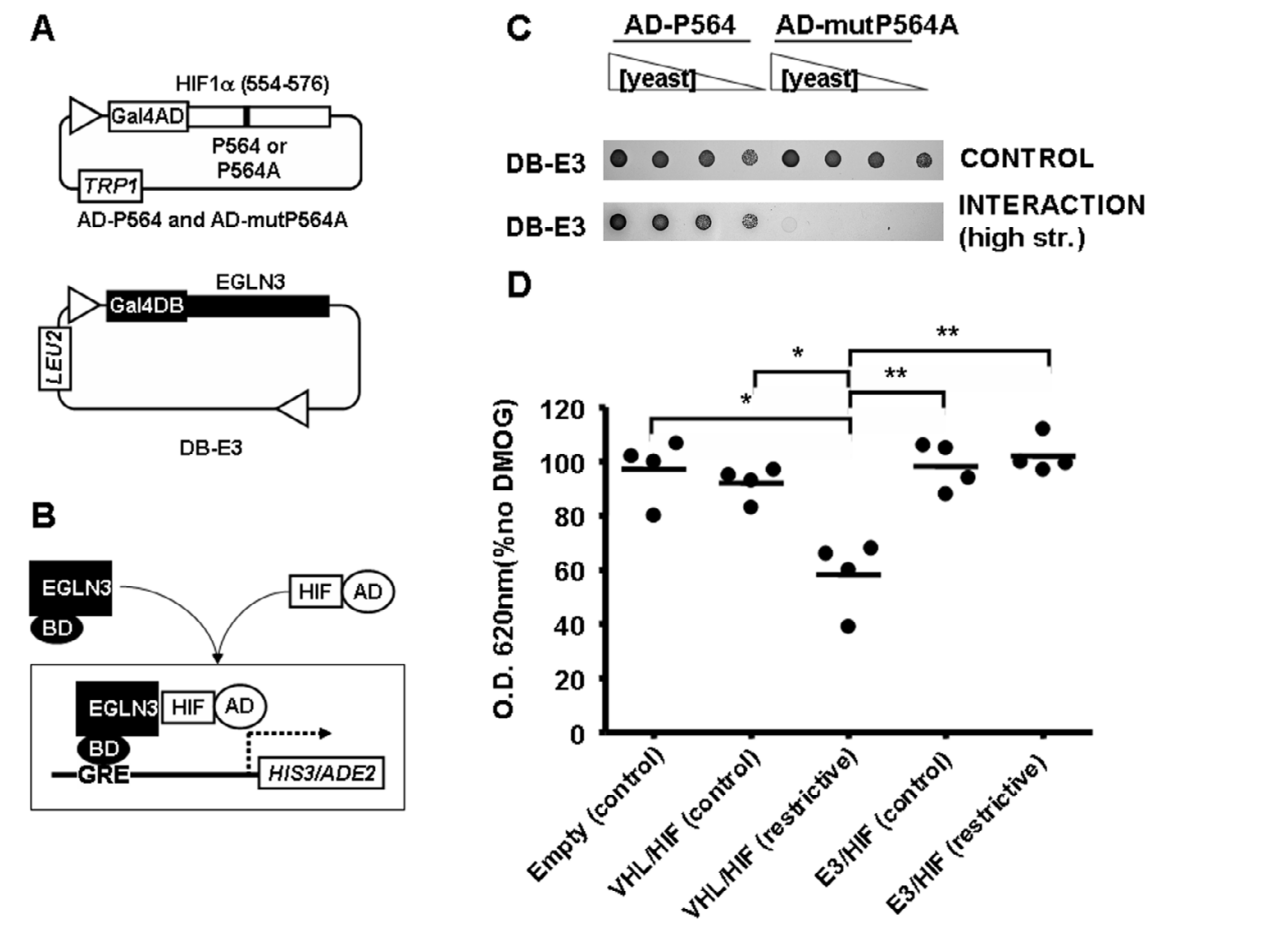
**Effect of DMOG on the direct binding of EGLN to HIF**. Schematic diagram representing the constructs (A) and the interaction between the different system components (B). C, Serial dilutions of clones transformed with the indicated constructs were grown on different stringency plates. The results shown are representative of at least three independent experiments. D, Yeast clones transformed with empty pGAD and pBridge plasmids (Empty) or with constructs encoding for AD-P564 and E3/DB-VHL (VHL/HIF) or AD-P564 and DB-E3 (E3/HIF) were used to inoculate control media lacking Leu and Trp (control) or restrictive media (restrictive) lacking Leu, Trp, and His (in the case of E3 clones restrictive media also lacked adenine). Duplicate cell cultures were grown in the presence of 0 or 500 μM DMOG and the density of cultures was determined 16–24 hours after inoculation. The density values obtained for cultures treated with DMOG were normalized as percentage of the density obtained for cultures in the absence of drug. The graph represents the individual data values of density for each of the four independent experiments. Horizontal bars represent the mean. Statistically significant different mean values are indicated with one (p < 0.05) or two (p < 0.01) asterisks. Symbols, abbreviations and panel labels are as in figure 1.

DMOG is a 2-oxoglutarate analogue that probably inhibits EGLN activity by competition with the reaction cosubstrate 2-oxoglutarate [42]. Thus, DMOG should affect EGLN catalitic activity (substrate hydroxylation), but not substrate binding. With the purpose of testing this hypothesis, we investigated the effect of DMOG on EGLN3 binding to HIF using a two hybrid-based assay [23]. In this assay, yeast growth in restrictive media is dependent on the direct interaction between a Gal-4 DNA binding domain-EGLN3 (DB-E3) and AD-HIF fusion proteins (figures 5A and 5B). The interaction between DB-E3 and AD-HIF is specific since a single mutation on the target proline in HIF sequence abolishes yeast growth (figure 5C) [23]. Importantly, this assay allows the direct comparison of DMOG effect on EGLN3 ability to bind HIF and act upon it. As shown in figure 5D, DMOG treatment had no effect on the direct binding of EGLN3 to its substrate, as demonstrated by the lack of effect on the growth of yeast expressing DB-EGLN3 and AD-HIF (E3/HIF system). However, at the same doses it clearly affected the hydroxylation of AD-HIF by EGLN3 (figure 5D, VHL/HIF system). This result indicates that, as expected, DMOG does not affect EGLN binding to substrate. Therefore, the effect of this drug on the reconstituted HIF system (figure 4) is likely due to its effect on EGLN activity rather than the EGLN3/HIF interaction. It should be noted however that these results do not exclude a potential effect of DMOG on VHL/hydroxylated-HIF interaction.

In addition to S956711 and DMOG, we also tested more general inhibitors, such as deferoxamine and cobalt chloride, that have been widely used to activate HIF [8,14]. EGLNs contain a tightly bound Fe^+2 ^atom in the catalitic site that is required as a cofactor in the hydroxylation reaction. Consequently, iron sequestration by the chelating agent deferoxamine results in EGLN inhibition [8,14]. On the other hand, the mechanism of EGLN inhibition by cobalt chloride is controversial [43,44]. It might act by substitution of the iron from the enzyme catalytic site and/or interfere with ascorbate transport [44], which is required to prevent the oxidation of the catalytic site of the enzyme. In our assays, cobalt chloride had an important toxic effect on yeast and showed little specific inhibition of the EGLN system (data not shown), thus we did not study it in further detail. On the other hand, we were unable to detect any significant effect of deferoxamine on yeast growth at concentrations up to 1 mM (data not shown). A possible explanation for the lack of effect of deferoxamine is that treatment might induce an adaptive response in yeast that increases their iron uptake [45]. In spite of their wide use as hypoxia mimetics, a recent report describes that cobalt and deferoxamine are ineffective inhibitors of EGLNs in vitro [43], which is in agreement with the lack of significant effect of these drugs in our system.

All together these results indicate that the combination of the control (Lck-dependent) and experimental (EGLN-dependent) systems constitutes an efficient assay to identify small molecules that have a specific effect on EGLN activity, while revealing those that have unspecific or toxic effects. Importantly, the effect of S956711 and DMOG in our system was achieved at doses equivalent to those required for the inhibition of EGLNs in mammalian cells [31,41].

## Discussion

Herein we describe a yeast three-hybrid system that reconstitutes HIF regulation through proline hydroxylation and VHL interaction. This system accurately preserves EGLN substrate specificity, supporting its biochemical relevance. The system described in this work relies on the interaction between a 22 residue fragment derived from HIF1α and the β-domain of VHL. The HIF1α fragment can be replaced by the full length protein without affecting the performance of system, except for a slight reduction in the interaction strength (data not shown). However, we were unable to express full length VHL in yeast (data not shown), probably due to misfolding of its α-domain in the absence of elongins B and C [46,47]. To circumvent this problem and since only the α domain of VHL interacts with elongin B [46], we generated a truncated VHL lacking this domain. This VHL form contains an intact β-domain [36] that, according to our data, is sufficient to bind HIF. Although the interaction of this truncated VHL construct with HIF is robust, we can not rule out that its affinity for HIF is different to that of the native VBC complex. In summary, this system is probably composed of the minimal functional units required to reconstitute HIF1α regulation.

Given the relevance of hypoxia in several pathologies, the identification of small molecules with the ability to modulate HIF activity has drawn much interest. In this regard, we have demonstrated that the three-hybrid assay described herein can be used to effectively identify small molecules that interfere with HIF regulation. Importantly, in contrast to current enzymatic assays, this system can be easily adapted to be performed on 96-well plates so it can be used for high-throughput screening of small molecules. In this regard, it should be noted that the direct determination of yeast growth provides a narrow linear range. Thus, in order to adapt this system to large high throughput screenings it would be much more convenient to determine β-galactosidase activity. Several yeast strains contain a *LacZ *gene under the control of the Gal4 promoter, thus β-galactosidase activity reflects the strength of the interaction between fusion proteins. In addition, the controls included in this work have proved useful in discriminating between genuine specific inhibitors of the EGLN-dependent system and molecules with a broad spectrum of targets such as cobalt chloride. Since the control (Lck-dependent assay) is based in exactly the same methodology than the test (EGLN-dependent assay), the combination of both systems constitutes a convenient assay for large screenings. Finally, comparison of the effects of the drug on the whole reconstituted system (three-hybrid) and on the direct interaction between EGLNs and HIFα (two-hybrid) could be helpful in the elucidation of the inhibition mechanism of candidate drugs (figure 5).

When considering the modulation of the HIF pathway, it is important to take into account that the relative contribution of each EGLN to HIF regulation seems to be different [17,22,23] and probably cell-type specific [21]. Moreover, it is likely that different EGLN isoforms might have a specific set of target molecules [24,25]. Thus, the development of isoform-specific inhibitors is of great interest. However, since the catalitic site of the three EGLNs members is very similar, the identification of specific inhibitors based on analogues of the 2-OG is probably difficult [48]. On the other hand, our work [49] and that of others [50] have recently demonstrated that the different substrate specificity shown by each EGLN isoform relies on a defined substrate-binding surface relatively far from the catalitic site. Thus, it is theoretically possible to identify molecules that block the HIF-binding region in specific EGLN isoforms. In this regard, use of our system, in particular the direct interaction assay (figure 5 and reference[23]), could aid in the identification/characterization of such a molecule.

During the writing of this manuscript a paper was published describing a yeast two-hybrid system that, similarly to ours, reconstitutes HIF recognition by VHL [51]. However, an important practical difference between the two systems is that the one described by Bex and coworkers required the coexpression of elongins in order to obtain a functional full length VHL.

## Conclusion

In summary, the results presented indicate that this system constitutes a simple yet powerful assay to study EGLN biochemistry in a cellular context without the interference of the endogenous HIF system. The system is particularly well suited to screen for modulators of the HIF regulatory machinery. Finally, since the specificity of the reaction catalyzed by the EGLNs is accurately preserved, this system can also be used to characterize novel EGLNs targets.

## Methods

### Cells and reagents

The *S. cerevisiae *AH109 strain, yeast growth media reagents YPD (yeast extract/peptone/dextrose), SD (synthetic defined) amino acids, X-gal (5-bromo-4-chloroindol-3-yl β-D-galactopyranoside) and plasmids (pGAD and pBridge) were from BD Biosciences, (Palo Alto, CA).

### Plasmid constructs

Constructs expressing HIF1α(554–576) and hSYK(11–263) as Gal4AD fusion proteins were generated by PCR amplification (figure 5 and reference [23]) of the indicated coding region from human cDNA and cloned into the EcoRI/BamHI or EcoRI sites of pGAD-T7 plasmid (BD Biosciences) respectively. The Gal4DBD fusion proteins were generated by PCR amplification (table 1) of the indicated coding regions from the cloned full length sequence (VHL) or from human cDNA (FceRIγ). VHL (encoding for residues 63–157) and FceRIγ (encoding for residues 45–86) amplicons were then cloned into the XmaI/SalI or EcoRI sites respectively. Finally, the coding sequences for EGLN3 and LCK were PCR-amplified (table 1) and cloned into the BglII or NotI/BglII sites of pBRIDGE-VHL(63–157) or pBRIDGE-FceRIγ (45–86) constructs respectively. LCK was amplified from human cDNA and EGLN-3 was amplified from its cloned cDNA, which was generously provided by Steven L. McKnight. For the two hybrid assays, the constructs expressing Gal4DBD fused to EGLN-3 were generated by cloning its coding sequence into the XmaI/SalI sites of the pBRIDGE plasmid (figure 5 and reference [23]).

**Table 1:** Primers used for the generation of the constructs used in this study.

Gene		Primer
Syk	Fw	5'GAATTCAACCACCTGCCCTTCTTT 3'
	Rv	5'GAATTCTGCCGATTTTTTGACATGG 3'
VHL	Fw	5'AGCTGAATTCCTGCGCTCGGTGAACTCGCGC3'
	Rv	5'AGCTCTCGAGGGATCCTCAAGTATACACTGGCAGTGTGATATTG3'
FcεRIγ	Fw	5'GAATTCCGACTGAAGATCCAAGTGCGAAAG 3'
	Rv	5'GAATTCCTACTGTGGTGGTTTCTCATGCTTCAG 3'
Lck	Fw	5'TGATCAGCGGCCGCCATGGGCTGTGGCTGCAGCTCACAC 3'
	Rv	5'GCGGCCGCTGATCATCAAGGCTGAGGCTGGTACTGGCC 3'
EGLN3	Fw	5'AGCTAAGCTTACCATGAGATCTTCTAGAATGCCCCTGGGACACATCATGAGG 3'
	Rv	5'GACTGAATTCTGATCAGTCGACGTCTTCAGTGAGGGCAGATTCAG 3'

In Table 2 are summed up all the constructs used in this study.

**Table 2:** Plasmids used in this study.

		Expression from PADH1 promoter			Expression from PMET25 promoter	
NAME	VECTOR	Protein	Fragment	MUTATIONS	Protein	Fragment

AD-P564	pGAD-T7	GAL4 AD- HIF	554–576	wt		
AD-mutP564A	pGAD-T7	GAL4 AD- HIF	554–576	P564A		
AD-L574A	pGAD-T7	GAL4 AD- HIF	554–576	L574A		
AD-L562R	pGAD-T7	GAL4 AD- HIF	554–576	L562R		
AD-P402	pGAD-T7	GAL4 AD- HIF	392–414	wt		
AD-SH2	pGAD-T7	GAL4 AD- SYK SH2	11–236	wt		
DB-VHL	pBRIDGE	GAL4 DBD- VHL	63–157	wt		
E3/DB-VHL	pBRIDGE	GAL4 DBD- VHL	63–157	wt	EGLN3	Full length
E1/DB-VHL	pBRIDGE	GAL4 DBD- VHL	63–157	wt	EGLN1	Full length
DB-ITAM	pBRIDGE	GAL4 DBD- ITAM	45–86	wt		
LCK/DB-ITAM	pBRIDGE	GAL4 DBD- ITAM	45–86	wt	LCK	Full length
DB-E3	pBRIDGE	GAL4 DBD- EGLN3	Full length	wt		

### Yeast transformation and plate interaction control assays

Freshly made competent yeast cells were transformed with 0.1 μg or 0.3 μg of GAL4AD or GAL4DBD constructs respectively by a modified version of the lithium acetate method (Gietz y col., 1992) and plated on minimal SD media plates in the absence of Leu and Trp for selection of transformants. Subsequently, an equal number of colonies from each transformation was transferred to saline solution (0.9% NaCl) and subjected to serial dilutions. Aliquots of each cell suspension (typically 8 μl) were plated on culture media with different stringencies: (1) control plates lacking leucine/tryptophan (minimal stringency; no interaction between fusion proteins required for yeast growth); (2) restrictive plates lacking leucine, tryptophan and histidine (medium stringency; interaction required to support yeast growth); or (3) restrictive plates lacking leucine, tryptophan, histidine and adenine (maximal stringency; strong interaction required to support yeast growth).

### Liquid media yeast growth assays

An equal number of fresh (up to two weeks old) yeast colonies were transferred to 6 ml of minimal stringency liquid media and grown overnight at 250 rpm and 30°C. Yeast cells were subsequently pelleted, thoroughly washed with maximal stringency medium and resuspended in 1 ml of the appropriate liquid culture media at a fixed concentration as determined in pilot experiments (Additional file 2). Cultures were then grown at 250 rpm and 30°C for the indicated times. Growth of cell cultures was measured as the increase in 620 nm absorbance of 150 μl culture aliquots taken at initial (t = 0) and the indicated times. For the drug assays, drugs were added to the culture media at t = 0.

### Statistical analysis of data

Experimental data were analyzed with the Prism™ GraphPad (version 4.01) software. Data were analyzed by the analysis of variance test (ANOVA) followed by the Tukey's multiple comparison test.

## List of abbreviations

HIF, hypoxia inducible factor; EGLN, *EGL*-nine Homolog; ODD, Oxygen-dependent Degradation Domain; AD, Gal4 activation domain; DB, Gal4 DNA binding domain; DMOG, dimethyloxaloylglycine.

## Authors' contributions

MA carried out all the experiments shown in figures 1, 2, 3, 4, 5 and Additional file 1. AVV generated the HIF/VHL/EGLN constructs for the two- and three-hybrid assays and did all the preliminary experiments for the hybrid assays. She also performed the experiments in Additional file 1. MOL and LFGF participated in the design and coordination of the study. LdP conceived the study, and participated in its design and coordination. He also performed the statistical analysis and wrote the manuscript. All authors read and approved the final manuscript.

## Supplementary Material

Additional file 1 **Characterization of the EGLN substrate specificity in the yeast three-hybrid system**. Yeast cells were treated as indicated in figure 1. AD-L574A and AD-L562R encode for the Gal-4 activation domain fused to HIF1α derived peptides expanding residues 554–576 and containing L574A or L562R mutations respectively. Mutation L574A prevents HIF hydroxylation by EGLNs [39]. In agreement, expression of none of the EGLN isoforms was able to trigger DB-VHL binding to AD-L574A in spite of the presence of an intact proline 564 residue. In contrast, mutation of L562R specifically affected the recognition of the substrate by EGLN3, while EGLN1 was unaffected. These results are in agreement with previous reports that indicate a preference of EGLN1, but not EGLN3, for arginine at that particular position [23,37]. The result shown is representative of two independent experiments. Click here for file

Additional file 2 **kinetics of growth in liquid media**. Yeast cells were transformed with constructs expressing AD-P564 and E3/DB-VHL or AD-SH2 and Lck/DB-ITAM. After transformation, cells were selected on plates lacking Leu and Trp. Individual transformed clones were harvested and resuspended in liquid media. The initial concentration of each yeast culture was adjusted in preliminary experiments so that all cultures achieved logarithmic growth in overlapping time windows. The figure shows the absolute densities of each culture at different time points after inoculation. In our assay conditions, the different cultures shared a wide time window (between 16 to 30 hours of growth) in which they all were in the exponential phase of growth. A representative experiment, of at least three independent, is shown. Click here for file
