# Supplementary figures and images for: A yeast three-hybrid system that reconstitutes mammalian hypoxia inducible factor regulatory machinery
Source: BMC Cell Biol. 2008 Apr 10;9:18. doi: 10.1186/1471-2121-9-18 (PMC2346465; doi:10.1186/1471-2121-9-18)

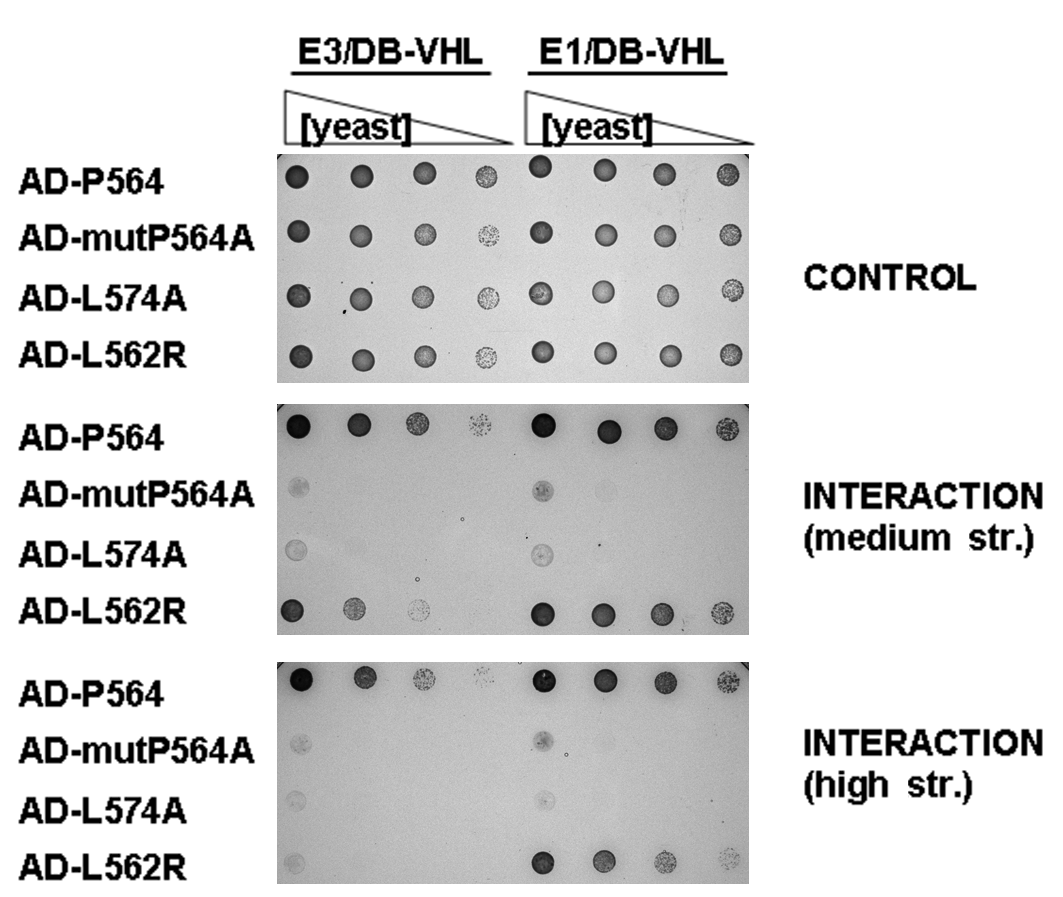

Supplement: Additional file 1 — Characterization of the EGLN substrate specificity in the yeast three-hybrid system. Yeast cells were treated as indicated in figure 1. AD-L574A and AD-L562R encode for the Gal-4 activation domain fused to HIF1α derived peptides expanding residues 554–576 and containing L574A or L562R mutations respectively. Mutation L574A prevents HIF hydroxylation by EGLNs [39]. In agreement, expression of none of the EGLN isoforms was able to trigger DB-VHL binding to AD-L574A in spite of the presence of an intact proline 564 residue. In contrast, mutation of L562R specifically affected the recognition of the substrate by EGLN3, while EGLN1 was unaffected. These results are in agreement with previous reports that indicate a preference of EGLN1, but not EGLN3, for arginine at that particular position [23,37]. The result shown is representative of two independent experiments. [file 1471-2121-9-18-S1.png]

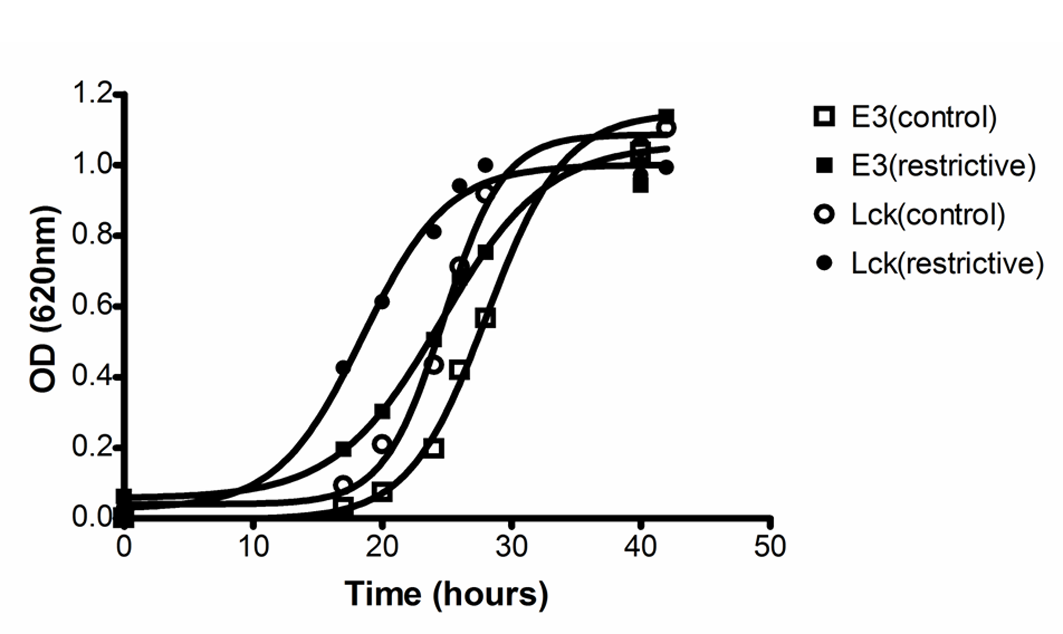

Supplement: Additional file 2 — kinetics of growth in liquid media. Yeast cells were transformed with constructs expressing AD-P564 and E3/DB-VHL or AD-SH2 and Lck/DB-ITAM. After transformation, cells were selected on plates lacking Leu and Trp. Individual transformed clones were harvested and resuspended in liquid media. The initial concentration of each yeast culture was adjusted in preliminary experiments so that all cultures achieved logarithmic growth in overlapping time windows. The figure shows the absolute densities of each culture at different time points after inoculation. In our assay conditions, the different cultures shared a wide time window (between 16 to 30 hours of growth) in which they all were in the exponential phase of growth. A representative experiment, of at least three independent, is shown. [file 1471-2121-9-18-S2.png]
